# Duchenne muscular dystrophy disease severity impacts skeletal muscle progenitor cells systemic delivery

**DOI:** 10.3389/fphys.2023.1190524

**Published:** 2023-05-09

**Authors:** Kholoud K. Saleh, Corey Switzler, Michael R. Hicks, Lily Gane, Devin E. Gibbs, April D. Pyle

**Affiliations:** ^1^ Department of Microbiology, Immunology and Molecular Genetics, University of California Los Angeles, Los Angeles, CA, United States; ^2^ Eli and Edythe Broad Center of Regenerative Medicine and Stem Cell Research, University of California Los Angeles, Los Angeles, CA, United States; ^3^ Molecular Biology Institute, University of California Los Angeles, Los Angeles, CA, United States

**Keywords:** skeletal muscle, systemic delivery, duchenne muscular dystrophy, skeletal muscle progenitor cells, intra-arterial cell delivery

## Abstract

Duchenne muscular dystrophy (DMD) is caused by an out-of-frame mutation in the DMD gene that results in the absence of a functional dystrophin protein, leading to a devastating progressive lethal muscle-wasting disease. Muscle stem cell-based therapy is a promising avenue for improving muscle regeneration. However, despite the efforts to deliver the optimal cell population to multiple muscles most efforts have failed. Here we describe a detailed optimized method of for the delivery of human skeletal muscle progenitor cells (SMPCs) to multiple hindlimb muscles in healthy, dystrophic and severely dystrophic mouse models. We show that systemic delivery is inefficient and is affected by the microenvironment. We found that significantly less human SMPCs were detected in healthy gastrocnemius muscle cross-sections, compared to both dystrophic and severely dystrophic gastrocnemius muscle. Human SMPCs were found to be detected inside blood vessels distinctly in healthy, dystrophic and severely dystrophic muscles, with prominent clotting identified in severely dystrophic muscles after intra arterial (IA) systemic cell delivery. We propose that muscle microenvironment and the severity of muscular dystrophy to an extent impacts the systemic delivery of SMPCs and that overall systemic stem cell delivery is not currently efficient or safe to be used in cell based therapies for DMD. This work extends our understanding of the severe nature of DMD, which should be taken into account when considering stem cell-based systemic delivery platforms.

## 1 Introduction

Duchenne Muscular Dystrophy (DMD) is a genetic disease characterized by progressive muscle degeneration and weakness due to the absence of dystrophin protein. Without dystrophin, the sarcolemma is rendered fragile and compromised causing the muscle fibers to go through progressive rounds of contraction-induced damage, and Ca^2+^ influx into the muscle fiber which results in cell death. In DMD, continuous cycles of contraction-induced damage elicit a constant need for regeneration. However, it has been suggested that there is impaired regeneration because either the muscle stem cell population, satellite cells (SCs), are rendered dysfunctional due to impaired polarity establishment or because of progressive exhaustion (Webster and Blau, 1990; Sacco et al., 2010; Dumont et al., 2015). Eventually, lack of proper regeneration leads to muscle fiber necrosis and generation of excess fibrotic tissue (Klingler et al., 2012).

Cell-based therapies were proposed for DMD by transplantation of myoblasts in which the enthusiasm of restoring dystrophin in the mdx mouse model by intra-muscular (IM) myoblast transplantation, resulted in 4 clinical trials in humans, that ultimately all failed to restore sufficient dystrophin to provide functional benefit (Partridge et al., 1989; Law et al., 1991; Gussoni et al., 1992; 1997; Huard et al., 1992; Karpati et al., 1993; Miller et al., 1997). The systemic delivery of various myogenic cell types to DMD muscles have been reported including mesoangioblasts, DLL4 and PDGF-BB treated satellite cells, skeletal muscle-derived CD133+ cells, and induced *Pax3* embryonic stem cell derived cells among others (Guttinger et al., 2006; Sampaolesi et al., 2006; Darabi et al., 2008; Tedesco et al., 2011; Sitzia et al., 2016; Gerli et al., 2019; Ausems et al., 2021). An overlooked parameter in developing systemic cell therapeutics is the context of disease severity and the diseased microenvironment. Genetic modifiers that regulate disease severity in the mdx mouse model is affected by the genetic background. Once the mdx mouse model is crossed to the DBA/2 genetic background, creating the mdxD2 strain, the mice exhibit increased fat and fibrosis deposition, muscle weakness, reduced skeletal muscle function, and fewer central myonuclei indicating the increased severity of dystrophy phenotype (Fukada et al., 2010; Coley et al., 2016). Thus, the mdxD2 mouse model better recapitulates the human disease and is useful in evaluating therapies for DMD. We have previously shown the complexity of muscle environment as DMD disease severity increases in mdx-NSG and mdxD2-NSG mice (Saleh et al., 2022). In this work, the mdx SCID mouse model was crossed with the severely immunocompromised mouse model NSG to generate mdx-NSG model. Moreover, the mdxD2 mouse was crossed with NSG to generate mdxD2-NSG mouse model. Mutations in the NSG mouse model renders B cells, T cells and natural killer cells deficient. These DMD severely immunocompromised mouse models are ideal for stem cell engraftment studies and cell delivery assessment as they permit evaluation of stem cell engraftment without the potential for immune-rejection of human cells.

Here we describe a detailed protocol to deliver human skeletal muscle progenitor cells (SMPCs) from fetal week 18 muscles to multiple hindlimb muscles in immunocompromised healthy, wt-NSG, dystrophic, mdx-NSG, and severely dystrophic, mdxD2-NSG, mouse models using intra-arterial (IA) delivery. This protocol has been optimized from other published protocols in two aspects, restoring blood flow into the artery after cell injection, and delivering the cells using a pump to maintain constant flow rate during cell delivery (Gerli et al., 2014; Matthias et al., 2015). We further investigated the detection and localization of SMPCs by quantifying the cells in at least 16 cross-sections taken across the length of the gastrocnemius muscles. We found overall that systemic delivery of SMPCs was inefficient in all models. However, significantly less human SMPCs were detected in wt-NSG gastrocnemius muscle cross-sections, compared to both mdx-NSG and mdxD2-NSG. Human SMPCs were found to be detected inside blood vessels distinctly in healthy, dystrophic and severely dystrophic muscles, with prominent clotting identified in mdxD2-NSG after IA systemic cell delivery. We propose that muscle microenvironment and the severity of muscular dystrophy to an extent impacts the systemic delivery of SMPCs and that overall systemic stem cell delivery is not currently efficient or safe to be used in cell based therapies for DMD.

## 2 Materials and methods

### 2.1 Institutional permissions

Human fetal muscle tissues were obtained from the University of California Los Angeles (UCLA) Center for AIDS Research (CFAR) Gene and Cellular Therapy Core and Advanced Bioscience Resources (ABR). Use of human tissues was institutional review board-approved by the UCLA Office of the Human Research Protection Program. Human fetal tissue work is IRB exempt. The work was reviewed and approved as exempt with IRB #20-000197 and IRB #15-000959. All animal work was conducted under protocols approved by the UCLA Animal Research Committee (ARC) (ARC-2006-119). Animals used in this study were housed in an immunocompromised core facility.

### 2.2 Animals

Animals used in this study were housed in UCLA Humanized Mouse Core, an immunocompromised core facility. C57BL/6 mice were crossed with NSG mice to generate C57-NSG mice (referred to as wt-NSG). mdx-NSG mice: mdx/C57BL/10 mice were crossed to NSG mice to generate mdx-NSG mice. mdxDBA2 mice were a generous gift from Dr. Melissa Spencer, UCLA, and were crossed to NSG mice to generate mdxD2-NSG mice. Pax7-Zsgreen mice were purchased from The Jackson Laboratory (#029549) and bred in house. Pups were genotyped using TransnetYX to ensure allele mutations. All animals used in this study were backcrossed to the original C57Bl/6 and mdxC57Bl/10 backgrounds for at least five generations.

### 2.3 Human muscle digestion

Muscles were first washed with 10% fetal bovine serum (FBS) in DMEM/F12% and 0.5% penicillin/streptomycin (P/S) for 10 min. The muscles were then finely chopped in digestion buffer consisting of 5% FBS in DMEM/F12, 0.5% P/S, 0.5% amphotericin (Ampho), 1 mg/ml collagenase II and 1 mg/ml dispase. The suspension was then incubated in 37°C on a shaker for 20–25 min, with intermittent trituration with 5 ml serological pipette. Once incubation time was over, 10% FBS wash buffer was added to the digested muscle to stop enzymatic digestion and the suspension spun at 2000 rpm at 10°C for 5 min. Then the supernatant was discarded and pellet was resuspended with sort buffer consisting of PBS -Mg^2+^/Ca^2+^, 1.5%–2% FBS and 0.5% P/S. The suspension was then passed through first 100 μm, then 70 µm and finally a 40 µm filters. The suspension was then spun at 2000 rpm at 10°C for 5 min and the pellet was resuspended with 5–6 ml of sort buffer. Cells were counted and blocked with Human TruStain FcX, then incubated with conjugated antibodies against CD31, CD235a, CD45, CD73, PDGFRα, CD11b and viability dye for 30 min. Lineage depleted cells were then sorted at UCLA Broad Stem Cell Research Center Flow Cytometry Core. Negatively depleted cells were then spun down at 1500 rpm for 5 min at room temperature, the pellet resuspended in SKGM-2 supplemented with 20 ng/ml bFGF and cultured overnight in 6 well plate, incubated for 60 min with matrigel (1 million counted cells/well). The cells were dissociated with TrypLE (Thermo Fisher) the following day for IA delivery or qRT-PCR quantification. Human fetal tissue was obtained from ABR and is IRB exempt. The work was reviewed and approved as exempt with IRB #20-000197.

### 2.4 Quantitative real time-PCR

Overnight cultured SMPCs were dissociated with TrypLE and RNA was extracted using the RNeasy Plus Micro Kit (Qiagen). cDNA was synthesized using the iScript Reverse Transcription Supermix (Bio-Rad) and quantitative RT-PCR was performed using SsoAdvanced Universal SYBR Green Supermix (Bio-Rad) with technical triplicates on a Thermo Fisher Scientific QuantStudio 6 Pro Real-Time PCR system or a CFX384 Touch Real-Time PCR Detection system (Bio-Rad). Primers used in this study are previously described in (Xi et al., 2017).

### 2.5 Intravenous cell delivery

Human SMPCs were isolated as previously described, with a change in the sorting strategy. ERBB3 and NGFR positive cells were sorted and propagated for a week before their IV tail vein injection. Mice were sacrificed 48 h after cell delivery.

### 2.6 Intra-arterial cell delivery protocol

Before cell delivery we included a 45-min downhill exercise regimen similar to described (Mathur et al., 2011) to induce muscle injury in the lower hindlimbs of mice before systemic SMPCs delivery. SMPCs can delivered through the femoral artery of either the left or right leg of the mouse. During the surgery, the mouse needs to be kept on a heating pad. All equipment used should be sterilized by wiping with 70% ethanol. Surgical tools need to be autoclaved the day before the surgery. A hot bead sterilizer was used to sterilize the surgical tools between mice. With an experienced hand, the surgery may take between 45 min to 1 h. The surgery should be done under the microscope in a biohazard BSL class II cabinet, to ensure the maintenance of sterility. It is important to prepare a cage with a heating pad under it to warm up the bedding for the recovering mouse after the surgery. Typically, 800K-1M cells were IA delivered/mouse. Cell dissociation was performed ahead the start of surgery of each mouse. The surgery was performed by making an incision at the inguinal region on the right hindlimb parallel to the femoral vascular bundle, and the femoral vascular bundle is exposed. The femoral artery is then isolated, two 6–0 sutures are passed under it, and one suture is used to obstruct blood flow, upstream of the injection site. In a proximal location to the body, a small cut is made in the femoral artery using a 32 G needle. A 32 G catheter is then inserted (cannulation site) into the femoral artery. Using a pump one million dissociated SMPCs (cultured overnight) are delivered at a flow rate of 50 ul/min in a volume of 100–150 μl of HBSS. After the cell injection, saline is flushed through the catheter to deliver any remaining cells in the catheter. The catheter then is retracted slowly, and while obstructing blood flow upstream of the injection site, a cautery is used to seal the femoral artery at the cannulation site. After sealing the femoral artery and removing the suture obstructing the blood flow, blood should be seen flushing again through the femoral artery. The opened incision area is filled with saline and the incision is sutured using a 5–0 absorbable suture. Mice are monitored after the surgery and kept in cages over a heating pad for recovery. Mice are provided with Carprofen 5 mg/kg of body weight. After 48 h mice were sacrificed and their lungs and muscles were collected and embedded in OCT and frozen in isopentane cooled in liquid nitrogen. Surgeries were performed on *n* = 3 mice/mouse model for human SMPCs delivery with about 800 K- 1 M cells delivered/mouse. For Zsgreen satellite cells delivery, cells were delivered after sorting and about 50k cells were delivered in each mouse.

### 2.7 Digital droplet PCR

Alternative sections were collected from muscles and lungs, and genomic DNA was isolated using the zymogen Quick DNA MiniPrep kit according to manufacturer instructions. Samples then were provided for the CFAR Virology Core Lab and Tissue Culture/PCR Facility for further ddPCR processing.

### 2.8 Immunofluorescence staining

Frozen muscles embedded in OCT were serially sectioned at 10 µm thick cryosections. A hydrophobic barrier was drawn around sections, then washed with 0.1% Tween in PBS (PBST). The sections were then fixed with 4% PFA for 10 min. A permeabilization step, if necessary, followed by washing with 0.3% Triton X-100 in PBS at room temperature for 10 min. Sections were then blocked with 0.25% Gelatin, 0.1% Tween, 3% bovine serum albumin (BSA) and 10% goat serum (GS) in distilled water for 60 min at room temperature. Sections were then incubated in humidified chambers with primary antibodies overnight at 4°C in 0.25% gelatin, 0.1% Tween, 3% BSA and 1% GS. Sections were next incubated for 60 min with fluorophore-conjugated secondary antibodies diluted in PBS and 1% goat serum. DAPI vecatshield mounting media was then used to counterstain nuclei, coverslips were applied and nail polish was used to seal the coverslips. Images were captured using a Zeiss Axio Observer. Z1 microscope equipped with an AxioCamMR3 camera.

### 2.9 Imaris quantification

At least 16–18 cross-sectional areas along the depth of the gastroc muscle were used for human cells (LaminA/C+ nuclei) quantification in each mouse (n = 3 wt-NSG, n = 3 mdx-NSG and n = 3 mdxD2-NSG) after IA cell delivery. Tile images (at 20X) of each cross-section were captured by Zeiss Axio Observer. Z1 microscope equipped with an AxioCamMR3 with Zen (2.6) blue edition. Zen files with Czi extension were converted and stitched in Imaris File Converter and Imaris Stitcher to an ims format. Images were then analyzed in Imaris software version 9.6 where each image included an endothelial cell marker (CD31-488), human cell marker (LaminA/C-568) and DAPI. Spots feature was used to quantify human cells on the 568 channel, surface feature was used to quantify blood vessels with areas >100 μm^2^ on the 488 channel, and then the object-object statistics was used to count the number of human cells inside specified blood vessels. Quantification is shown as mean + SD, One way ANOVA with Tukey’s multiple comparisons was used to compare the means of cells detected inside large vessels in wt-NSG, mdx-NSG and mdxD2-NSG.

## 3 Results

### 3.1 Optimized intra-arterial cell delivery protocol to lower hindlimb muscles

**FIGURE 1:**
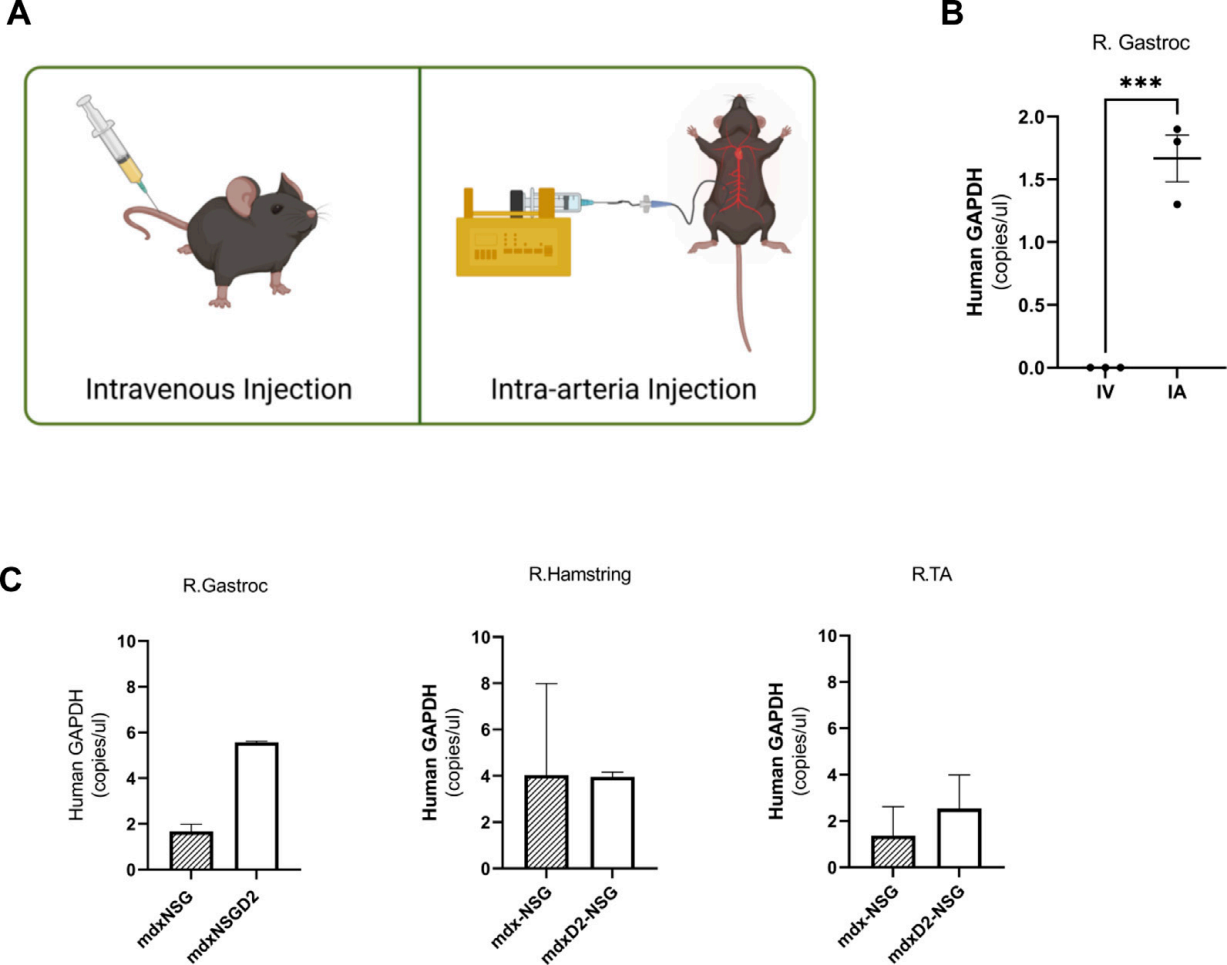
Intra-arterial cell delivery route improves delivery of human skeletal muscle progenitor cells to lower hindlimb gastrocnemius muscle. **(A)** Intravenous (IV) injection is performed by tail vein injection while intra-arterial (IA) cell delivery is performed by injecting the cells into the femoral artery of either the right or left hindlimb. **(B)** Human specific-GAPDH concentration measured in homogenized cross sections in mdx-NSG right gastrocnemius muscle (R. Gastroc) after IV and IA delivery utilizing digital droplet polymerase chain reaction. Alternative sections were collected for gDNA isolation, error bars showing mean ± SD. **(C)** Human specific-GAPDH concentration measured in homogenized cross sections of mdx-NSG and mdxD2-NSG right hindlimb muscles (where IA surgery was performed). Error bars showing mean + SD.

**FIGURE 2:**
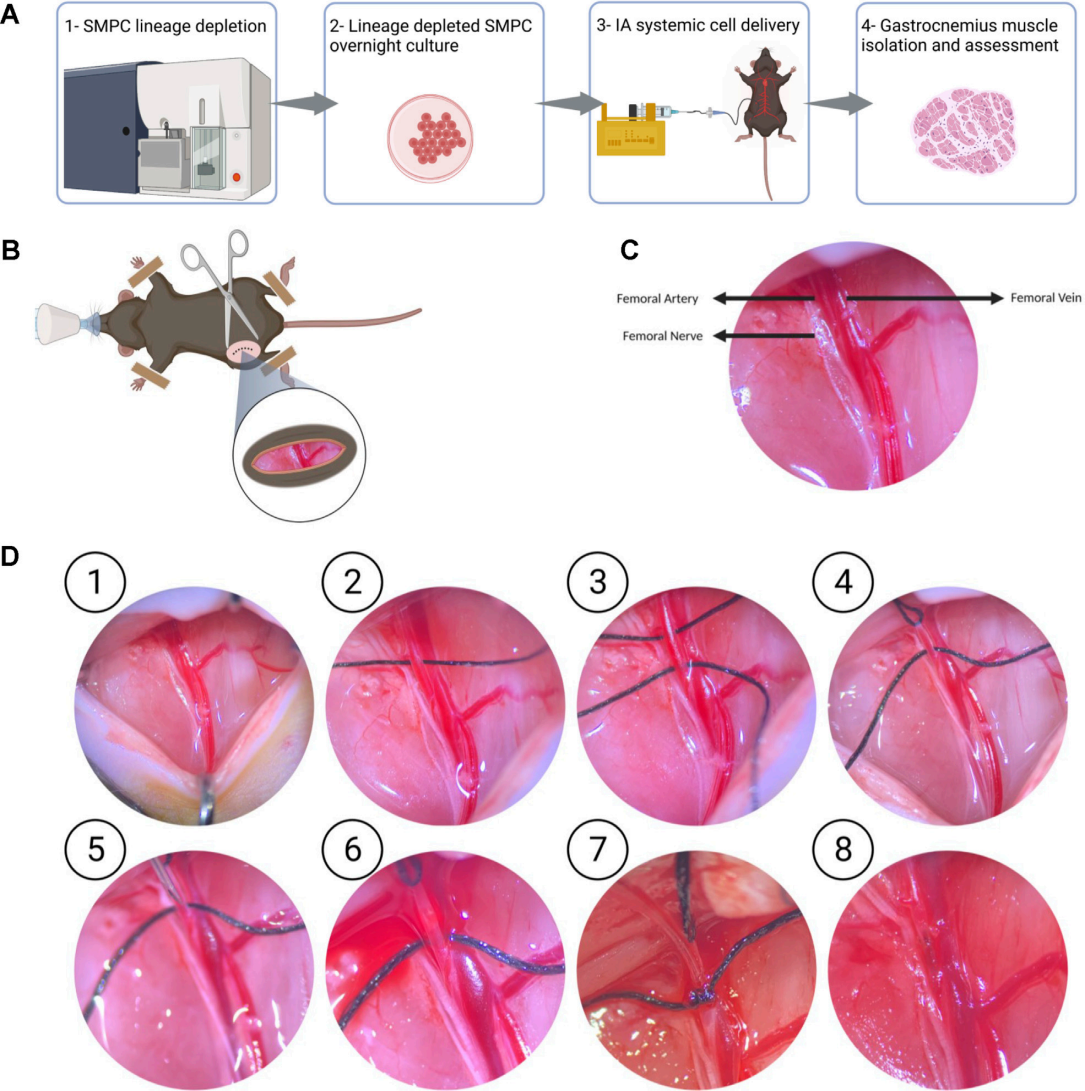
Optimized intra-arterial cell delivery protocol to lower hindlimb muscles **(A)** Schematic of experimental flow of IA systemic cell delivery. FW18/19 are lineage depleted and cultured overnight, cells are dissociated, and IA delivered at a 50 µl/min flow rate, and lower hindlimb muscles, including gastrocnemius muscle, are collected 48 h after surgery. **(B, C)** The femoral bundle consisting of the femoral artery, femoral nerve and femoral vein are exposed once retractors are used to open the incision site. **(D)** Detailed protocol of intra-arterial cell delivery where ① the femoral nerve and femoral artery are dissected away from the femoral vein. ② and ③ Two 6–0 size silk sutures, are passed underneath the femoral artery. ④ the silk suture upstream of the injection site is pulled to stop blood flow. ⑤ and ⑥ A 30 G needle is used to make a small puncture in the femoral artery downstream of the blood occlusion site ⑦ 32G catheter is then inserted into the femoral artery and secured with the second suture downstream of the injection site. Cell infusion is started at 50 μl/min ⑧ After untying the suture securing the catheter and retrieving the catheter from the femoral artery, a cauterizer is used to seal the infusion site. After removing the suture occluding the blood flow to the femoral artery, blood flow should be seen restored into the femoral artery.

Lineage depleted SMPCs derived from FW 18 muscles were differentiated *in vitro* for 5 days in N2 media to validate their myogenic potential (Supplementary Figures S1A, B). We explored two routes of delivery of human SMPCs to lower hindlimb muscles, the first is an intravenous route (IV) where cells were injected into the tail vain of mice, and the second is intra-arterial (IA) where cells were delivered at a flow to the femoral artery (Figure 1A). We evaluated SMPC delivery by measuring human-specific GAPDH in muscle cross-sections using digital droplet polymerase chain reaction (ddPCR). Muscles were serially sectioned, at 10 μm thick cryosections, across the entire muscles with alternative sections collected for ddPCR. Human specific-GAPDH was detected in gastrocnemius muscles with IA delivered cells, but not IV delivered cells (Figure 1B) in mdx-NSG mice. Because cells were not detected in the gastrocnemius muscle after IV delivery in mdx-NSG mouse, we focused our efforts in optimizing the IA delivery method in mdx-NSG and mdxD2-NSG mice. We were able to detect human-specific GAPDH in the right hindlimb muscles, where surgery is performed, of mdx-NSG and mdxD2-NSG mice 48 h after cell delivery (Figure 1C), but not in the contralateral hindlimb (data not shown). In intramuscular (IM) cell engraftment experiments, muscles are typically injured 24 h before cell delivery using cardiotoxin, barium chloride, or cryoinjury, to induce muscle damage and assist in cell engraftment (Brimah et al., 2004; Ehrhardt et al., 2007; Darabi et al., 2008; Sacco et al., 2010; Sakai et al., 2013; Hicks et al., 2018). As this is not feasible for systemic delivery, we included a 45-min downhill exercise regimen similar to described to induce muscle injury in the lower hindlimbs of mice before systemic SMPC delivery (Mathur et al., 2011). Matthias et al. (2015) described a detailed method for IA cell delivery through the femoral artery with successful detection of human cells in the muscle. However, in the previous protocol after cell delivery the femoral artery is ligated, which in our hands caused a prominent ischemia injury detected in the gastrocnemius muscle of mdx-NSG mice (Supplementary Figure S1C). Femoral artery ligation has been demonstrated to cause ischemic gastrocnemius muscle injury with a decreased blood flow to the limb below the ligation site (Paoni et al., 2002; Padgett et al., 2016; Tu et al., 2021). Another IA cell delivery protocol described by Gerli et al. (2014) re-establishes blood flow in the femoral artery after cell delivery, however, the cells are not delivered at a constant flow rate. Our approach is optimized from other IA delivery strategies by combining both delivery of the cells at a constant flow rate of 50 μl/min of cell suspension using a catheter, and by restoring blood flow after cell delivery, to prevent muscle ischemic injury (Figures 2A–D). Approximately 800K-1M SMPCs were delivered to male wt-NSG, mdx-NSG or mdxD2-NSG mice.

### 3.2 The severity of DMD mouse model impacts systemic human skeletal muscle progenitor cells delivery into lower hindlimb muscles

**FIGURE 3:**
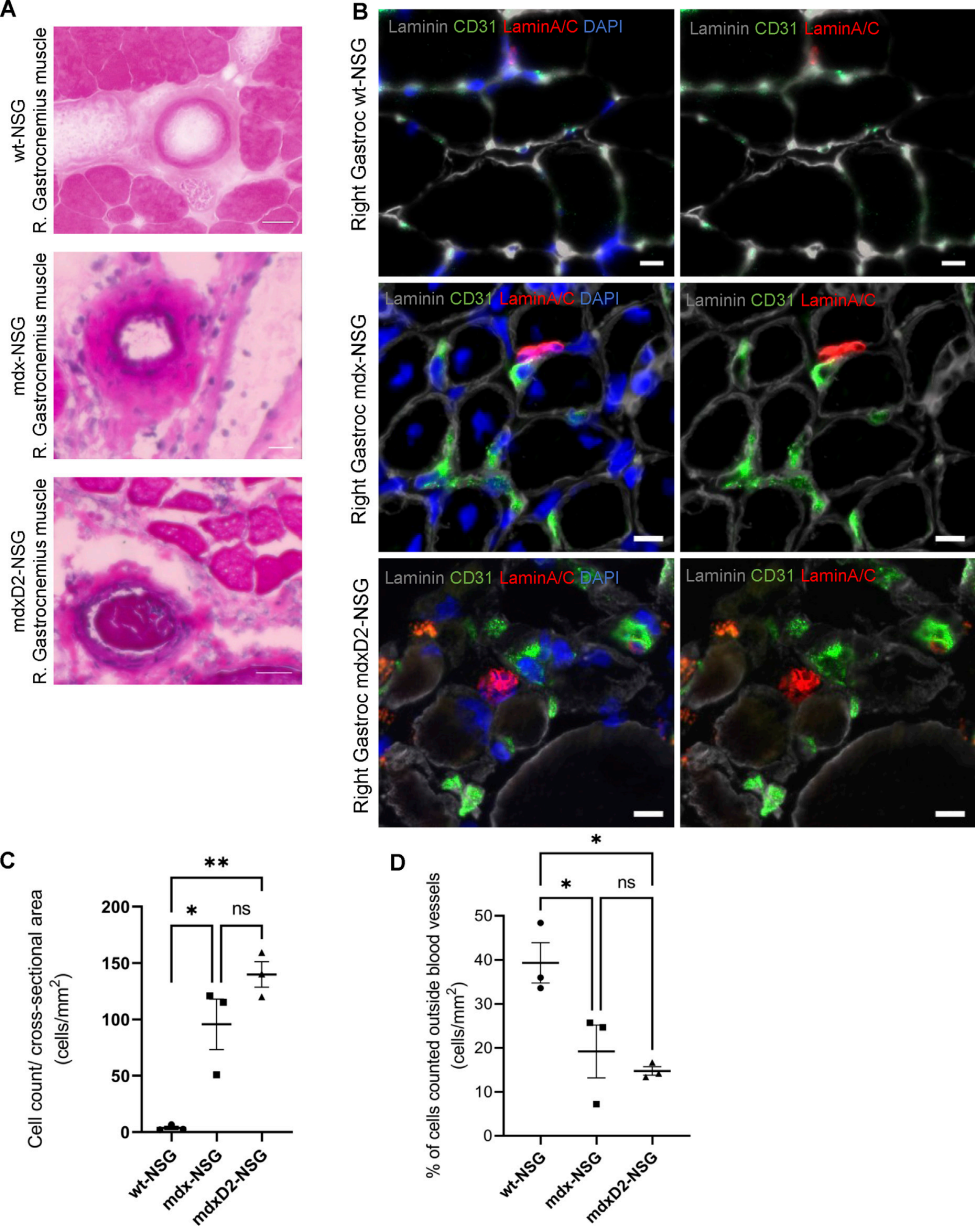
The severity of DMD mouse model impacts systemic human skeletal muscle progenitor cells delivery into lower hindlimb muscles. **(A)** Histological analysis of right gastrocnemius muscle after intra-arterial cell delivery in wt-NSG, mdx-NSG and mdxD2-NSG muscles. **(B)** Gastrocnemius muscle cross-sections staining for human skeletal muscle progenitors cells (marked by human nuclie marker LaminA/C, red) and blood vessels (marked by endothelial cells marker CD31, green) with human cells detected outside the blood vessels. Scale bars at 10 µm. **(C)** Plot of the total human cells quantified/cross-sectional area of the gastrocnemius muscles of wt-NSG, mdx-NSG and mdxD2-NSG mouse models. (*N* = 3/mouse model, One Way ANOVA with Tukey's multiple comparisons, errors bars show mean ± SD, *p ≤ 0.01, **p << 0.01). **(D)** Plot of the percentage of the total cells quantified outside blood vessels/cross-sectional area in wt-NSG, mdx-NSG and mdxD2-NSG muscles. (*N* = 3/mouse model, One Way ANOVA with Tukey’s multiple comparisons, errors bars show mean±SD, *p ≤ 0.05).

Despite our efforts to prevent muscle injury after optimization of IA systemic delivery procedure, severe injury was detected in the lower limb muscles in mdxD2-NSG mouse model, but not in mdx-NSG or wt-NSG (Supplementary Figure S2). To further investigate this finding, we performed histological analysis using hematoxylin and eosin staining on gastrocnemius muscle cross-sections of wt-NSG, mdx-NSG and mdxD2-NSG after SMPCs IA delivery. We observed prominent clotting occurring in large blood vessels in mdxD2-NSG, but not in wt-NSG and mdx-NSG (Figure 3A). These findings indicate a pronounced difference in SMPC IA cell delivery in severely dystrophic mouse model compared to healthy and dystrophic mouse models. To evaluate SMPC localization after their IA systemic delivery and whether the cells home to the muscle, right gastrocnemius muscles cross-sections were analyzed from all mouse models. In a successful cell delivery procedure, the human cells are expected to be detected outside the blood vessels and homing to the muscle (Figure 3B). Using both Zen 2.6 and Imaris Cell Imaging software we quantified human LaminA/C cells, a human cell perinuclear marker, in at least 16 cross-sections taken across the length of the muscle. Interestingly, we found significantly more human cells detected in gastrocnemius muscles per total cross-sectional area quantified in mdx-NSG and mdxD2-NSG compared to wt-NSG (Figure 3C). Of the total counted cells, significantly higher number of SMPCs, about 40%, quantified in wt-NSG gastrocnemius muscle cross-sections were detected outside the blood vessels, while only about an average of 20% and 18% were detected outside blood vessels in mdx-NSG and mdxD2-NSG, respectively (Figure 3D).

**FIGURE 4:**
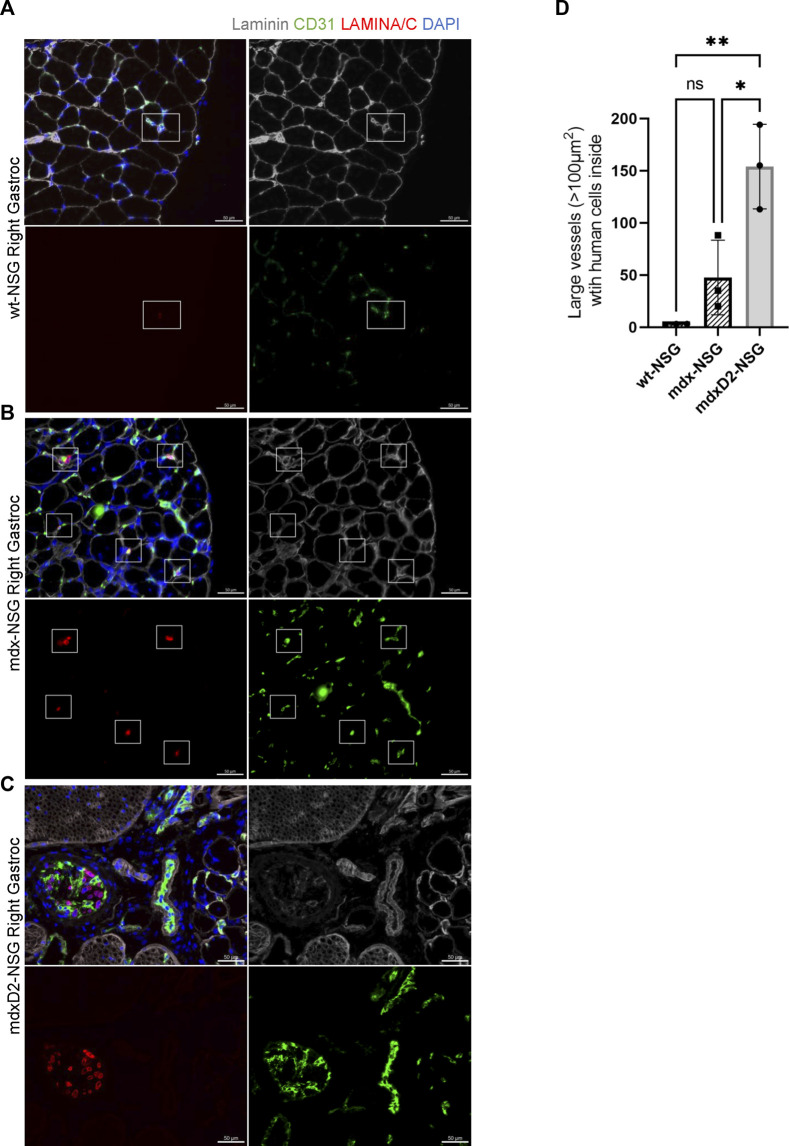
Significantly more human skeletal muscle progenitor cells are detected in large blood vessels in severely dystrophic gastrocnemius muscles. **(A–C)** Cross sections of right gastrocnemius muscles of wt-NSG, mdx-NSG, and mdxD2-NSG showing human nuclei (H-laminA/C, red) 48 h post IA cell delivery. SMPCs are detected inside blood vessels. Scale bars at 50 µm. **(D)** Comparison of the average number of blood vessels with a cross-sectional area >100 μm^2^ with SMPCs detected inside in wt-NSG, mdx-NSG and mdxD2-NSG gastrocnemius muscles. (One way ANOVA with Tukey’s multiple comparisons, error bars represent mean ± SD, *p < 0.05, **p < 0.01).

Because of the high frequency of detecting human SMPCs inside blood vessels, we then focused on evaluating the localization of SMPCs within blood vessels (Figures 4A–C). On average of the total SMPCs quantified in all gastrocnemius muscle cross-sections, 60%, 84%, and 62% of cells were observed inside the smallest blood vessel unit, the capillaries, in wt-NSG, mdx-NSG and mdxD2-NSG, respectively (not shown). We next sought to determine the differences in cell localization in large blood vessels between wt-NSG, mdx-NSG and mdxD2-NSG gastrocnemius muscles. Using Imaris Cell Imaging software we measured the area of larger vessels (veins/venules and arteries/arterioles) with human cells inside them and found that in mdx-NSG gastrocnemius muscles an average of 74 blood vessels with cross-sectional areas greater than 100 μm^2^ had SMPCs detected in them, compared to an average of 154 blood vessels in mdxD2-NSG (Figure 4D). SMPCs detected inside 100 μm^2^ blood vessel or greater in wt-NSG was negligible. Taken together we found that human SMPCs engraft inefficiently to lower hindlimb healthy and dystrophic muscles and are mainly detected inside blood vessels. We observed that human SMPCs localize in blood vessels distinctly in wt-NSG and mdx-NSG and mdxD2- NSG, with prominent clotting identified in mdxD2-NSG after human SMPCs IA systemic delivery. These findings indicate that the severity of DMD impacts human SMPC systemic delivery.

**FIGURE 5:**
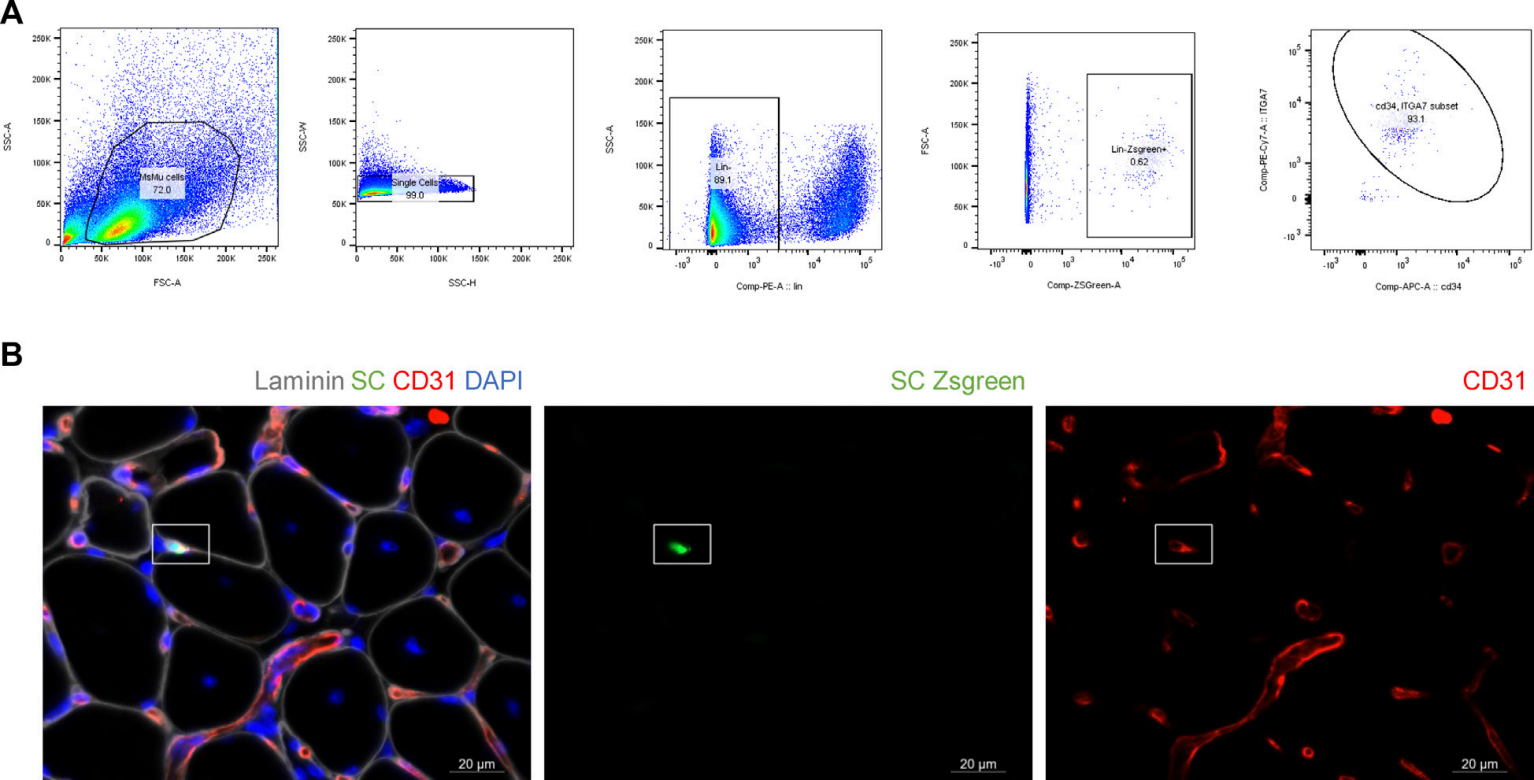
Mouse satellite cells do not efficiently extravasate. **(A)** Isolation of Zsgreen positive satellite cells for IA delivery in mdx-NSG mouse model. To confirm that Zsgreen cells isolated are indeed satellite cells, CD34 and ITGα7 staining was also performed on ZsGreen cells. Of the total Zsgreen + cells analyzed, 93% were CD34 and ITGα7 positive. **(B)** 50 K of healthy mouse Zsgreen satellite cells (green) were IA delivered to mdx-NSG mice (n = 2) and they were detected in capillaries. Scale bar 20 µm.

To verify that the previous findings were not influenced by cell size of human SMPCs, we IA delivered SCs to mdx-NSG mice (n = 2). For easier detection of the mouse SCs after delivery, we used a Pax7-ZsGreen transgenic mouse model that express enhanced green fluorescent protein for SCs isolation (Figure 5A). Because SCs are fewer in adult mouse muscle tissue, only about 50 K Zsgreen + SCs were delivered after their sort. The Zsgreen SCs were still detected inside capillaries in the mdx-NSG model, suggesting that the size of human SMPCs is not a main factor for their detection in capillaries (Figure 4B). This finding also suggests that human SMPC and mouse SCs are equally not equipped with the machinery that would enable them to home to the muscle.

## Discussion

Cell based therapies for muscle diseases including DMD offer enormous potential for personalized therapies especially in combination with gene correction (Young et al., 2016; Hicks et al., 2018). The challenge faced is the lack of the ability to efficiently deliver cells to multiple muscles, which will be needed for neuromuscular diseases where multiple muscles are affected. Previously published reports for systemic delivery targeting multiple muscles did not deliver cells at a constant flow rate or were not able to re-establish blood flow back into the artery after cell delivery. Therefore, it was imperative to optimize an IA cell delivery protocol that provides delivered cells with the optimal conditions for muscle homing. Here we developed an optimized protocol for IA cell delivery by delivering the cells at a constant flow rate using a pump and catheter, and by establishing blood flow back into the femoral artery after cell delivery. This protocol confirmed its versatility with SMPCs delivered in three different mouse models, immunocompromised healthy and two dystrophic mouse models. Although this protocol has been developed for the delivery of SMPCs, it can be adapted for the delivery of other cell types, which we demonstrated by the delivery of mouse SCs, or for the delivery of modalities important for gene therapies.

The optimization of the IA protocol led us to focus on evaluating the systemic delivery potential of SMPCs in healthy and DMD mouse models. We found that although SMPCs can reach multiple lower limb muscles after IA systemic delivery, the efficiency is too low to lead to robust long-term engraftment. Moreover, cells are detected in the tibialis anterior, gastrocnemius, hamstring and lateral thigh muscles of the injected leg (right or left) where the procedure is performed, but not in the contralateral hindlimb muscle (data not shown). Evaluation of gastrocnemius muscle structure after IA delivery showed severe clotting evident in mdxD2-NSG, but not in wt-NSG or mdx-NSG muscles. Despite the fact that about the same number of cells were delivered to all mice across the mouse models, significantly fewer human SMPCs were detected in the wt-NSG gastrocnemius muscles in overall quantified cross-sectional areas. Interestingly, of the total human SMPCs quantified in the wt-NSG gastrocnemius muscle cross-sections, significantly higher number of cells were detected outside blood vessels compared to mdx-NSG and mdxD2-NSG. We therefore propose that less human SMPCs adhered to blood vessels in wt-NSG muscles compared to both DMD mouse models, which indicates a role the diseased microenvironment plays in the efficiency of systemic cell delivery.

Nonetheless, our current findings do not suggest that human SMPCs are homing efficiently to healthy muscle, as the majority of human SMPCs quantified were still detected inside blood vessels in all mouse models. However, interestingly, the localization of human SMPCs was distinct between the mouse models, with cells in the mdxD2-NSG observed in large blood vessels forming clots, not observed in mdx-NSG and wt-NSG muscles. These findings suggest that SMPCs are not endowed with the machinery to escape blood vessels to the surrounding muscle. Due to technical limitations, low number of SCs were delivered to the hindlimb muscle of mdx-NSG mice. However, as a proof of concept, we have shown that the gold standard mouse muscle stem cells also do not have the potential to extravasate efficiently. This challenge could perhaps be overcome by overexpressing the components needed to enable cells to extravasate utilizing the machinery used by leukocytes (Ley et al., 2007), but will require extensive optimization and a combination of cell and gene therapy approaches to overcome this large barrier to systemic based deliveries to muscle.

In summary, here we optimized an IA cell delivery protocol that can be utilized for both cell delivery and potentially combination cell and gene therapy applications. We have shown that IA systemic based cell delivery can be performed using human SMPCs, but the efficiency is too low to be considered for use in therapeutic applications. Indeed, in severely dystrophic mouse models it could be detrimental. We have shown differences in human SMPCs delivery in healthy, dystrophic and severely dystrophic muscles. These findings highlight the need to further understand the differences in skeletal muscle microenvironment between healthy, dystrophic and severely dystrophic mouse models, namely, endothelial cells that line the interior surface of blood vessels. Future studies will likely need to develop combination therapies targeting both the diseased microenvironment as well as generating a better SMPC that can be engineered to extravasate in parallel prior to use in regenerative medicine approaches for muscular dystrophy.

## Data Availability

The original contributions presented in the study are included in the article/Supplementary Material, further inquiries can be directed to the corresponding author.

## Supplementary material

Supplementary Figure 1 Optimized intra-arterial cell delivery prevents severe muscle ischemia injury **(A)** qRT-PCR of myogenic markers of lineage depleted human skeletal muscle progenitor cells (FW18). *n* = 3 biological samples, data are normalized to GAPDH as mean ± SD of technical triplicates. **(B)** Lineage depleted human skeletal muscle progenitor cells (FW18) fuse and form PAX7+ cells when differentiated *in vitro* for 5 days in N2 media. Scale bar at 100 µm. **(C)** Histological analysis, hematoxylin and eosin, staining of mdx-NSG gastrocnemius muscle 48 h after intra-arterial cells delivery in the right femoral artery. Top: control left gastrocnemius muscle, bottom left: mdx-NSG right gastrocnemius muscle with femoral artery ligation, bottom right: right gastrocnemius muscle with intra-arterial cell delivery procedure optimization fibrotic areas.

Supplementary Figure 2 Lower hindlimb morphology after intra-arterial cell delivery in mdx-NSG and mdxD2-NSG mice. mdxD2-NSG muscles show severe ischemia with H&E staining showing blood clots inside large blood vessels whereas control hindlimbs do not.

## References

[B1] Ausems C. R. M. Engelen B. G. M. V. Bokhoven H. Wansink D. G. (2021). Systemic cell therapy for muscular dystrophies. Stem Cell Rev. Rep. 17, 878–899. 10.1007/s12015-020-10100-y 33349909 PMC8166694

[B2] Coley W. D. Bogdanik L. Vila M. C. Yu Q. Meulen J. H. V. D. Rayavarapu S. (2016). Effect of genetic background on the dystrophic phenotype in mdx mice. Hum. Mol. Genet. 25, 130–145. 10.1093/hmg/ddv460 26566673 PMC4690497

[B3] Darabi R. Gehlbach K. Bachoo R. M. Kamath S. Osawa M. Kamm K. E. (2008). Functional skeletal muscle regeneration from differentiating embryonic stem cells. Nat. Med. 14, 134–143. 10.1038/nm1705 18204461

[B4] Dumont N. A. Wang Y. X. Maltzahn J. Pasut A. Bentzinger C. F. Brun C. E. (2015). Dystrophin expression in muscle stem cells regulates their polarity and asymmetric division. Nat. Med. 21, 1455–1463. 10.1038/nm.3990 26569381 PMC4839960

[B5] Fukada S. Morikawa D. Yamamoto Y. Yoshida T. Sumie N. Yamaguchi M. (2010). Genetic background affects properties of satellite cells and mdx phenotypes. Am. J. Pathol. 176, 2414–2424. 10.2353/ajpath.2010.090887 20304955 PMC2861106

[B6] Gerli M. F. M. Maffioletti S. M. Millet Q. Tedesco F. S. (2014). Transplantation of induced pluripotent stem cell-derived mesoangioblast-like myogenic progenitors in mouse models of muscle regeneration. J. Vis. Exp. Jove, e50532. 10.3791/50532 24472871 PMC4089411

[B7] Gerli M. F. M. Moyle L. A. Benedetti S. Ferrari G. Ucuncu E. Ragazzi M. (2019). Combined notch and PDGF signaling enhances migration and expression of stem cell markers while inducing perivascular cell features in muscle satellite cells. Stem Cell Rep. 12, 461–473. 10.1016/j.stemcr.2019.01.007 PMC6409426 30745033

[B8] Gussoni E. Blau H. M. Kunkel L. M. (1997). The fate of individual myoblasts after transplantation into muscles of DMD patients. Nat. Med. 3, 970–977. 10.1038/nm0997-970 9288722

[B9] Gussoni E. Pavlath G. K. Lanctot A. M. Sharma K. R. Miller R. G. Steinman L. (1992). Normal dystrophin transcripts detected in Duchenne muscular dystrophy patients after myoblast transplantation. Nature 356, 435–438. 10.1038/356435a0 1557125

[B10] Guttinger M. Tafi E. Battaglia M. Coletta M. Cossu G. (2006). Allogeneic mesoangioblasts give rise to alpha-sarcoglycan expressing fibers when transplanted into dystrophic mice. Exp. Cell Res. 312, 3872–3879. 10.1016/j.yexcr.2006.08.012 16982052

[B11] Hicks M. R. Hiserodt J. Paras K. Fujiwara W. Eskin A. Jan M. (2018). ERBB3 and NGFR mark a distinct skeletal muscle progenitor cell in human development and hPSCs. Nat. Cell Biol. 20, 46–57. 10.1038/s41556-017-0010-2 29255171 PMC5962356

[B12] Huard J. Bouchard J. P. Roy R. Malouin F. Dansereau G. Labrecque C. (1992). Human myoblast transplantation: Preliminary results of 4 cases. Muscle Nerve 15, 550–560. 10.1002/mus.880150504 1584246

[B13] Karpati G. Ajdukovic D. Arnold D. Gledhill R. B. Guttmann R. Holland P. (1993). Myoblast transfer in duchenne muscular dystrophy. Ann. Neurol. 34, 8–17. 10.1002/ana.410340105 8517684

[B14] Klingler W. Jurkat-Rott K. Lehmann-Horn F. Schleip R. (2012). The role of fibrosis in Duchenne muscular dystrophy. Acta Myol. Myopathies Cardiomyopathies Off. J. Mediterr. Soc. Myol. 31, 184–195. PMC3631802 23620650

[B15] Law P. K. Goodwin T. G. Fang Q. Chen M. Li H. Florendo J. A. (1991). Myoblast transfer therapy for duchenne muscular dystrophy. Pediatr. Int. 33, 206–215. 10.1111/j.1442-200x.1991.tb01545.x 1957647

[B16] Ley K. Laudanna C. Cybulsky M. I. Nourshargh S. (2007). Getting to the site of inflammation: The leukocyte adhesion cascade updated. Nat. Rev. Immunol. 7, 678–689. 10.1038/nri2156 17717539

[B17] Mathur S. Vohra R. S. Germain S. A. Forbes S. Bryant N. D. Vandenborne K. (2011). Changes in muscle T2 and tissue damage after downhill running in mdx Mice. Muscle Nerve 43, 878–886. 10.1002/mus.21986 21488051 PMC3101319

[B18] Matthias N. Hunt S. D. Wu J. Darabi R. (2015). Skeletal muscle perfusion and stem cell delivery in muscle disorders using intra-femoral artery canulation in mice. Exp. Cell Res. 339, 103–111. 10.1016/j.yexcr.2015.08.018 26341268

[B19] Miller R. G. Sharma K. R. Pavlath G. K. Gussoni E. Mynhier M. Yu P. (1997). Myoblast implantation in Duchenne muscular dystrophy: The San Francisco study. Muscle Nerve 20, 469–478. 10.1002/(sici)1097-4598(199704)20:4<469::aid-mus10>3.0.co;2-u 9121505

[B20] Partridge T. A. Morgan J. E. Coulton G. R. Hoffman E. P. Kunkel L. M. (1989). Conversion of mdx myofibres from dystrophin-negative to -positive by injection of normal myoblasts. Nature 337, 176–179. 10.1038/337176a0 2643055

[B21] Sacco A. Mourkioti F. Tran R. Choi J. Llewellyn M. Kraft P. (2010). Short telomeres and stem cell exhaustion model duchenne muscular dystrophy in mdx/mTR mice. Cell 143, 1059–1071. 10.1016/j.cell.2010.11.039 21145579 PMC3025608

[B22] Saleh K. K. Xi H. Switzler C. Skuratovsky E. Romero M. A. Chien P. (2022). Single cell sequencing maps skeletal muscle cellular diversity as disease severity increases in dystrophic mouse models. Iscience 25, 105415. 10.1016/j.isci.2022.105415 36388984 PMC9646951

[B23] Sampaolesi M. Blot S. D’Antona G. Granger N. Tonlorenzi R. Innocenzi A. (2006). Mesoangioblast stem cells ameliorate muscle function in dystrophic dogs. Nature 444, 574–579. 10.1038/nature05282 17108972

[B24] Sitzia C. Farini A. Jardim L. Razini P. Belicchi M. Cassinelli L. (2016). Adaptive immune response impairs the efficacy of autologous transplantation of engineered stem cells in dystrophic dogs. Mol. Ther. 24, 1949–1964. –1964. 10.1038/mt.2016.163 27506452 PMC5154479

[B25] Tedesco F. S. Hoshiya H. D’Antona G. Gerli M. F. M. Messina G. Antonini S. (2011). Stem cell–mediated transfer of a human artificial chromosome ameliorates muscular dystrophy. Sci. Transl. Med. 3, 96ra78. 10.1126/scitranslmed.3002342 21849666

[B26] Webster C. Blau H. M. (1990). Accelerated age-related decline in replicative life-span of Duchenne muscular dystrophy myoblasts: Implications for cell and gene therapy. Somat. Cell Molec Gen. 16, 557–565. 10.1007/bf01233096 2267630

[B27] Xi H. Fujiwara W. Gonzalez K. Jan M. Liebscher S. Handel B. V. (2017). *In vivo* human somitogenesis guides somite development from hPSCs. Cell Rep. 18, 1573–1585. 10.1016/j.celrep.2017.01.040 28178531 PMC5327729

[B28] Young C. S. Hicks M. R. Ermolova N. V. Nakano H. Jan M. Younesi S. (2016). A single CRISPR-cas9 deletion strategy that targets the majority of DMD patients restores dystrophin function in hiPSC-derived muscle cells. Cell Stem Cell 18, 533–540. 10.1016/j.stem.2016.01.021 26877224 PMC4826286

