# Supplementary material for: Duchenne muscular dystrophy disease severity impacts skeletal muscle progenitor cells systemic delivery
Source: Front Physiol. 2023 May 9;14:1190524. doi: 10.3389/fphys.2023.1190524 (PMC10203213; doi:10.3389/fphys.2023.1190524)
Supplement: Supplementary file 1 [file Table1.docx]

# Resource Identification Initiative

| **REAGENT or RESOURCE** | **SOURCE** | | **RRID or CATALOGUE NUMBER** | |
| --- | --- | --- | --- | --- |
| Anti Human CD45-PE | Thermo Fisher Scientific | | Cat# 12-0459-42, RRID: AB_1724079 | |
| Anti Human CD11b-PE | Biolegend | | Cat# 301306, RRID: AB_314158 | |
| Anti-Human CD235a - PE | Thermo Fisher Scientific | | Cat# 12-9987-82,  RRID: AB_466300 | |
| Anti-Human PDGFRα-PE | BD Biosciences | | Cat# 556002,  RRID:AB_396286 | |
| Anti-Human CD73 | BD Biosciences | | Cat# 550257,  RRID:AB_393561 | |
| Anti-Human CD31 (PECAM-1)-PE | Thermo Fisher Scientific | | Cat# 12-0319-42,  RRID: RRID:AB_10669160 | |
| Dead Cell Stain Kit | Thermo Fisher Scientific | | Cat# L34975, | |
| Anti-Mouse CD31 (MEC 13.3) | BD Biosciences | | Cat# 553370,  RRID: AB_394816 | |
| Anti-Mouse Laminin | Sigma-Aldrich | | Cat# L9393,  RRID:AB_477163 | |
| Lamin A/C Monoclonal Antibody (mab636) | Thermo Fisher Scientific | | Cat# MA3-1000,  RRID:AB_325377 | |
| Anti-Rat Alexa fluor 488 | Fisher Scientific | | Cat# A11006, RRID:AB_2534074 | |
| Anti-Rabbit Alexa Fluor 647 | Fisher Scientific | | Cat# A21245,  AB_2535813 | |
| Anti-Mouse IgG2b Fluor 568 | Fisher Scientific | | Cat# A21144,  RRID:AB_2535780 | |
| DMEM/F-12, HEPES medium | Thermo Fisher Scientific | | Cat# 11330032 | |
| DPBS | Gibco | Cat#14190-136 | |  |
| Collagenase, Type 2 (Collagenase II) | Worthington-Biochem | | Cat# LS004177 | |
| Dispase II | Thermo Fisher Scientific | | Cat# 17105041 | |
| Amphotericin B | Thermo Fisher Scientific | | Cat# 15290018 | |
| Fetal bovine serum (FBS) | Thermo Fisher Scientific | | Cat# 16000044 | |
| SkGM-2 Skeletal Muscle Cell Growth Medium-2 BulletKit | Lonza | | Cat# CC-3245 | |
| TrypLE Express | Thermo Fisher Scientific | | Cat# 12605010 | |
| Human bFGF | Proteintech | | Cat# HZ-1285 | |
| Hank’s Buffered Salt Solution | Thermo Fisher Scientific | | Cat# 14175095 | |
| Matrigel | Corning | | Cat#354277 | |
| SkGM™-2 Skeletal Muscle Cell Growth Medium-2 BulletKit™ | Lonza | | Cat#CC-3245 | |
| AO/PI | Nexcelom | |  | |
| Silk Suture, Size 6-0 | Fisher Scientific | | Cat#50-118-0809 | |
| Ethicon Vicryl Suture, absorbable, Size 5-0 | Fisher Scientific | | Cat#NC9335902 | |
| 32 G femoral artery catheter-Mouse (Recathco) | Fisher Scientific | | Cat#50-196-383 | |
| Butterfly Needles | SAI infusion technologies | | Sku#BFL-24 | |
| Disposable Hypodermic Needles (30G) (Exel) | Fisher Scientific | | Cat#14-841-03 | |
| High Temperature Cautery Kit | Fine Science Tools | | Cat#18010-00 | |
| **Biological samples** |  | |  | |
| Human muscle tissues of fetal week 17/18 | University of California Los Angeles (UCLA) Center for AIDS Research (CFAR) Gene and Cellular Therapy Core and Advanced Bioscience Material | | N/A | |
| **Experimental models: Organisms/strains** |  |  |  |  |
| C57Bl/6-NSG (wt-NSG) | N/A | | N/A | |
| mdx-NSG | N/A | | N/A | |
| mdxD2-NSG | N/A | | N/A | |
| **Software** |  |  |  |  |
| Zen 2.6 (blue edition) | Carl Zeiss Microscopy | |  | |
| IMARIS Version 9.6 | OXFORD Instruments | | <http://www.bitplane.com/imaris/imaris> | |
| Prism 9.1.1 | GraphPad | | [https://www.graphpad.com](https://www.graphpad.com/) | |
