# Supplementary figures and images for: Duchenne muscular dystrophy disease severity impacts skeletal muscle progenitor cells systemic delivery
Source: Front Physiol. 2023 May 9;14:1190524. doi: 10.3389/fphys.2023.1190524 (PMC10203213; doi:10.3389/fphys.2023.1190524)

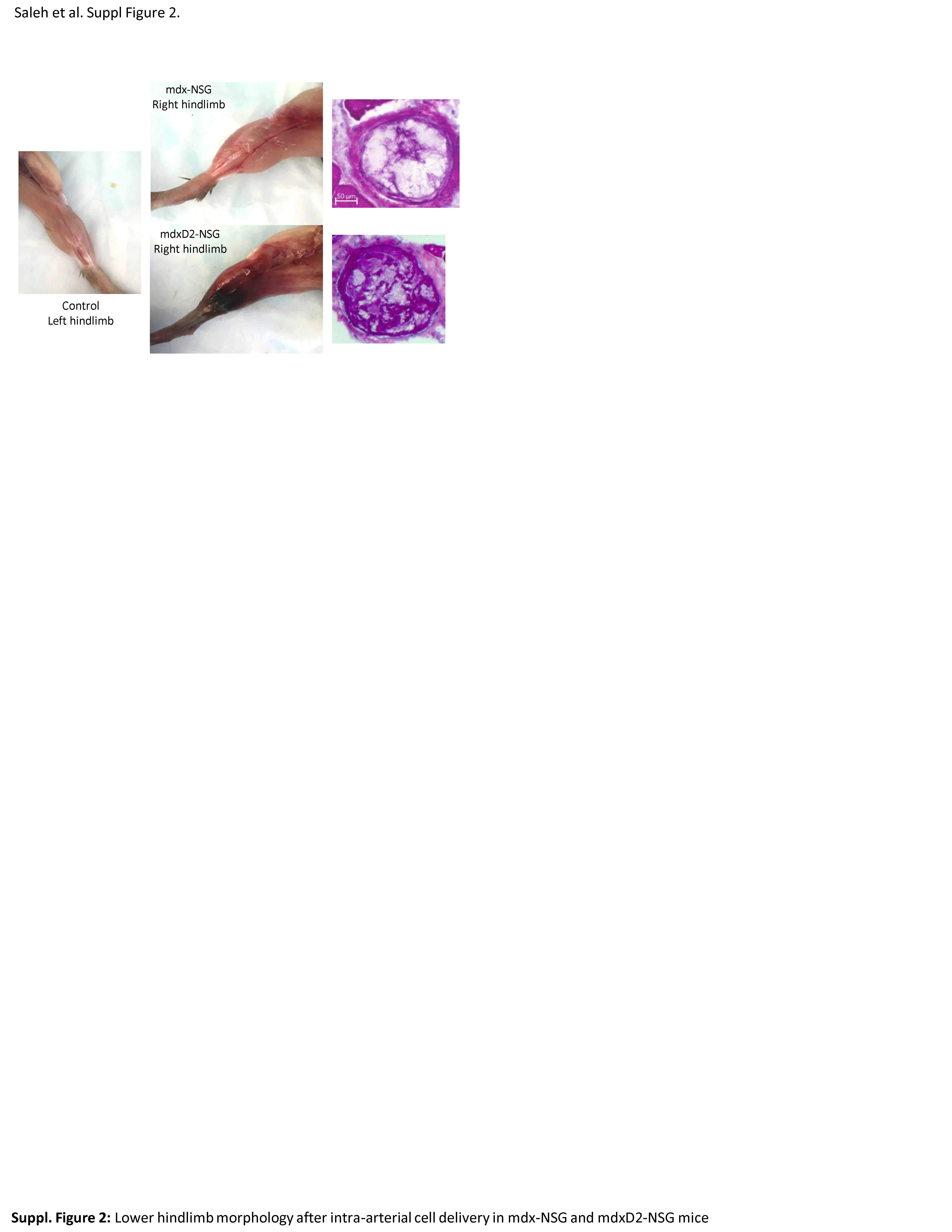

Supplement: Supplementary file 2 [file Image2.tif]

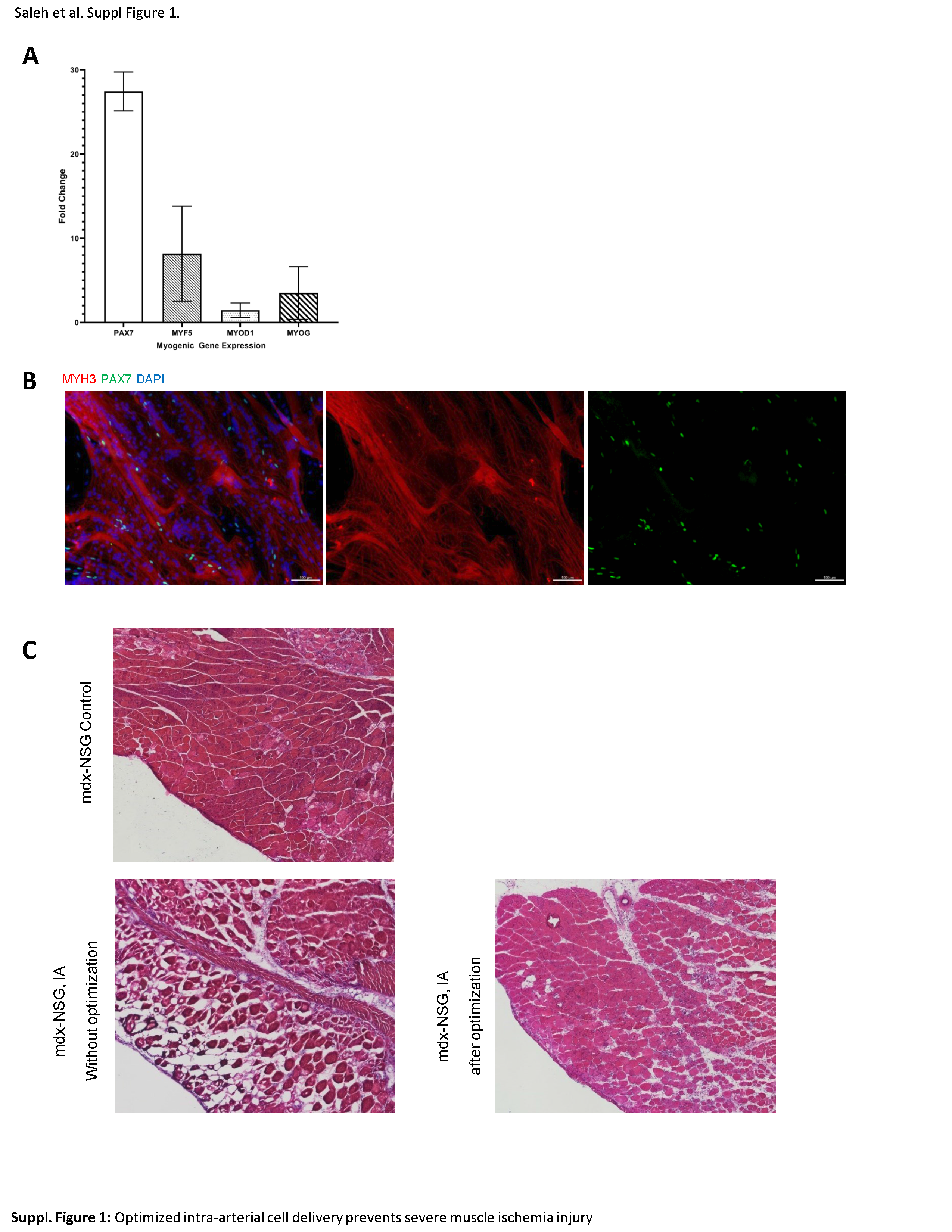

Supplement: Supplementary file 3 [file Image1.tif]
