# Supplementary material for: Duchenne muscular dystrophy disease severity impacts skeletal muscle progenitor cells systemic delivery
Source: Front Physiol. 2023 May 9;14:1190524. doi: 10.3389/fphys.2023.1190524 (PMC10203213; doi:10.3389/fphys.2023.1190524)
Supplement: Supplementary file 4 [file DataSheet1.docx]

**Supplemental Figure 1: Optimized intra-arterial cell delivery prevents severe muscle ischemia injury**

A) qRT-PCR of myogenic markers of lineage depleted human skeletal muscle progenitor cells (FW18). n=3 biological samples, data are normalized to GAPDH as mean ± SD of technical triplicates.

B) Lineage depleted human skeletal muscle progenitor cells (FW18) fuse and form PAX7+ cells when differentiated in vitro for 5 days in N2 media. Scale bar at 100 µm.

C) Histological analysis, hematoxylin and eosin, staining of mdx-NSG gastrocnemius muscle 48 hours after intra-arterial cells delivery in the right femoral artery. Top: control left gastrocnemius muscle, bottom left: mdx-NSG right gastrocnemius muscle with femoral artery ligation, bottom right: right gastrocnemius muscle with intra-arterial cell delivery procedure optimization fibrotic areas.

**Supplemental Figure 2: Lower hindlimb morphology after intra-arterial cell delivery in mdx-NSG and mdxD2-NSG mice.**

mdxD2-NSG muscles show severe ischemia with H&E staining showing blood clots inside large blood vessels whereas control hindlimbs do not.
